# In vitro Multi-targeted Anti-cancer Effects of Bavachinin in Papillary Thyroid Carcinoma Cell Line: Dual Pathway Inhibition and Cytokine Downregulation

**DOI:** 10.5812/ijpr-166894

**Published:** 2025-11-27

**Authors:** Gang Duan, Zahra Zahid Piracha, Umar Saeed, Yanrong Tian

**Affiliations:** 1 Provincial Endemic Diseases Laboratory, Shaanxi Provincial Center for Disease Control and Prevention, Xi'an, China; 2 Faculty of Rehabilitation and Allied Health Sciences, Riphah International University, Islamabad, Pakistan; 3 Institute of Graduate Studies and Research, Cyprus International University, Nicosia, Cyprus; 4 Szechenyi Istvan University, Gyor, Hungary; 5 University College, Korea University, Seoul 02418, Republic of KOREA (South KOREA); 6 Department of Anesthesiology, Xi'an International Medical Center Hospital, Xi'an, China

**Keywords:** Thyroid Neoplasms, Flavonoids, Bavachinin, Apoptosis, Signal Transduction, Cytokines

## Abstract

**Background:**

Thyroid cancer is the most common endocrine malignancy, with aggressive subtypes frequently demonstrating resistance to conventional therapies. Bavachinin, a natural flavonoid derived from *Psoralea corylifolia*, has exhibited anti-cancer activity in various tumor models; however, its effects on thyroid cancer remain largely undefined.

**Objectives:**

The aim of this study is to evaluate the anti-cancer activity of bavachinin in the papillary thyroid carcinoma TPC-1 cell line and elucidate its underlying molecular mechanisms.

**Methods:**

TPC-1 cells were treated with bavachinin (5 - 20 μM) for 24 - 72 hours. Cell viability was assessed using the 3-(4,5-Dimethylthiazol-2-yl)-2,5-diphenyltetrazolium bromide (MTT assay); morphological changes were visualized by confocal microscopy. Migration and invasion were analyzed by wound-healing and Transwell assays, respectively. Cytokine secretion was measured using enzyme-linked immunosorbent assay (ELISA). Gene and protein expression levels of protein kinase B (AKT), mechanistic target of rapamycin (mTOR), extracellular signal-regulated kinase 1/2 (ERK1/2), and c-Jun N-terminal kinase (JNK) were evaluated by quantitative real-time polymerase chain reaction (qRT-PCR) and Western blotting. Apoptosis was confirmed by assessing the B-cell lymphoma-2-associated X protein (BAX)/B-cell lymphoma-2 (BCL-2) ratio and cleaved caspase-3 activity. All experiments were performed in triplicate, and data are presented as mean ± standard deviation (SD). Statistical significance was determined by one-way analysis of variance (ANOVA) followed by Tukey’s post-hoc test (P < 0.05).

**Results:**

Bavachinin significantly reduced cell viability, migration, and invasion in a dose-dependent manner (20 μM reduced viability by approximately 50% at 72 hours, P < 0.01). It suppressed the phosphorylation of AKT and ERK1/2, downregulated mTOR expression, and decreased secretion of tumor necrosis factor-alpha (TNF-α) and interleukin-1 beta (IL-1β). Apoptosis was confirmed by an increased BAX/BCL-2 ratio and elevated cleaved caspase-3 levels.

**Conclusions:**

Bavachinin exerts multi-targeted anti-cancer effects in thyroid carcinoma cells through dual inhibition of the phosphoinositide-3-kinase (PI3K)/AKT/mTOR and mitogen-activated protein kinase (MAPK)/ERK pathways, along with suppression of pro-inflammatory cytokines, culminating in apoptosis and impaired invasiveness.

## 1. Background

Thyroid cancer is the most prevalent endocrine malignancy, with global incidence continuing to rise (1). Although most cases are associated with a favorable prognosis, aggressive subtypes remain challenging to treat due to therapeutic resistance and frequent recurrence (2), underscoring the urgent need for novel agents that can suppress proliferation and metastasis.

The phosphoinositide-3-kinase (PI3K)/protein kinase B (AKT)/mechanistic target of rapamycin (mTOR) and mitogen-activated protein kinase (MAPK)/extracellular signal-regulated kinase 1/2 (ERK1/2) cascades are pivotal drivers of thyroid tumor growth, survival, and metastasis (3). These pathways promote cell proliferation and inhibit apoptosis — PI3K/AKT primarily through mTOR-mediated protein synthesis and MAPK/ERK via BRAF- or RAS-driven activation (4-6). Crosstalk between these pathways amplifies oncogenic signaling and contributes to resistance against therapy. Concurrently, pro-inflammatory cytokines such as tumor necrosis factor-alpha (TNF-α) and interleukin-1 beta (IL-1β) further stimulate tumorigenesis, thereby highlighting inflammation as a critical therapeutic target (1, 6, 7).

Apoptosis via the intrinsic mitochondrial pathway, characterized by increased B-cell lymphoma-2-associated X protein (BAX), reduced B-cell lymphoma-2 (BCL-2), and activation of caspase-3, is a widely recognized mechanism of effective anti-cancer agents (8, 9). Furthermore, inhibition of cell migration and invasion is essential for preventing metastasis (10).

Bavachinin, a natural flavonoid with antioxidant and anti-inflammatory properties, has demonstrated anti-tumor activity in other cancers (11, 12). However, its effects on thyroid cancer and its impact on these key signaling pathways remain unexplored.

To address this gap, we examined the anti-cancer effects of bavachinin in TPC-1 papillary thyroid carcinoma cells. We assessed cell viability (MTT assay), morphology (confocal microscopy), and migration/invasion (wound-healing and Transwell assays). Mechanistic insights were obtained from quantitative real-time polymerase chain reaction (qRT-PCR) analysis of AKT, mTOR, ERK1/2, and N-terminal kinase (JNK), along with Western blotting and enzyme-linked immunosorbent assay (ELISA) to evaluate protein and cytokine levels. Apoptosis was assessed by evaluating the BAX/BCL-2 ratio and cleaved caspase-3 expression.

## 2. Objectives

Although flavonoids are known to target cancer pathways, studies specifically investigating bavachinin in thyroid carcinoma are lacking. Here, we demonstrate that bavachinin suppresses cell viability, migration, and invasion while promoting apoptosis through modulation of the BAX/BCL-2 axis and activation of caspase-3. Mechanistically, it inhibits the PI3K/AKT/mTOR and MAPK/ERK signaling pathways and downregulates pro-inflammatory cytokines, supporting its potential as a multi-targeted therapeutic candidate for papillary thyroid carcinoma.

## 3. Methods

### 3.1. Reagents and Materials

Bavachinin (≥ 95% purity, Sigma-Aldrich, Cat. No. SMB00100) was dissolved in dimethyl sulfoxide (DMSO), with a final DMSO concentration ≤ 0.1% in all treatments. LY294002 (PI3K inhibitor, Cell Signaling Technology, Cat. No. 9901) and U0126 (MEK inhibitor, Cell Signaling Technology, Cat. No. 9903) were used for pathway inhibition. Roswell Park Memorial Institute (RPMI)-1640 medium (Gibco, Cat. No. 11875-093), fetal bovine serum (FBS; Gibco, Cat. No. 10082147), and penicillin-streptomycin (Gibco, Cat. No. 15140-122) were used for cell culture. All chemicals were of analytical grade.

### 3.2. Cell Culture and Treatments

The human papillary thyroid carcinoma cell line TPC-1 was obtained from the American Type Culture Collection (ATCC). Cells were cultured in RPMI-1640 medium supplemented with 10% FBS and 1% penicillin-streptomycin at 37°C in a humidified 5% CO_2_ atmosphere. Bavachinin (dissolved in DMSO, final DMSO ≤ 0.1%) was administered at concentrations of 5, 10, and 20 μM for 24, 48, or 72 hours. For pathway inhibition experiments, cells were pre-treated for 1 hour with LY294002 (20 μM, PI3K inhibitor) or U0126 (20 μM, MEK inhibitor) prior to bavachinin exposure.

Untreated (vehicle-only, DMSO ≤ 0.1%) TPC-1 cells served as experimental controls in all assays, while LY294002 and U0126 were used as pathway-specific positive controls for PI3K and MAPK inhibition, respectively. All experiments were independently performed in triplicate (n = 3), and data are presented as mean ± standard deviation (SD) to ensure reproducibility and statistical robustness.

Preliminary MTT assays determined a 48-hour half-maximal inhibitory concentration (IC_50_) of approximately 22 - 24 μM for bavachinin in TPC-1 cells; therefore, 20 μM was selected as the upper treatment concentration to approximate but not exceed the IC_50_, allowing evaluation of sub-IC_50_ (5 and 10 μM) responses.

### 3.3. MTT Cell Viability Assay

Cell viability was assessed using the MTT assay. Absorbance was measured at 570 nm and normalized to control (100%). The bavachinin concentrations (5, 10, and 20 μM) for viability testing were selected following preliminary cytotoxicity screening and are supported by published data showing significant anti-cancer activity of bavachinin at 10 - 30 μM in non-small cell lung cancer cell lines (13).

### 3.4. Confocal Microscopy

Cells were fixed, permeabilized, and stained with DAPI (blue) and phalloidin-Alexa Fluor 594 (red) as previously described (14). Images were acquired using a confocal microscope (e.g., Zeiss LSM 700).

### 3.5. Wound Healing Assay

A linear scratch was made in confluent TPC-1 monolayers, which were then treated with bavachinin in serum-free medium for 24 hours. Images at 0 and 24 hours were analyzed for percentage wound closure.

### 3.6. Transwell Invasion Assay

Transwell inserts (8 μm pore size, Corning, Cat. No. 3422) coated with Matrigel (Corning, Cat. No. 354234) were used to assess cell invasiveness. After 24 hours of incubation, non-invaded cells on the upper surface were removed with a cotton swab; invaded cells on the lower surface were fixed with 4% paraformaldehyde, stained with crystal violet (Sigma-Aldrich, Cat. No. C0775), and counted under a light microscope.

### 3.7. Quantitative Real-time Polymerase Chain Reaction Analysis

Total RNA was extracted and converted to complementary DNA (cDNA) for qRT-PCR using SYBR Green Master Mix (Applied Biosystems) and gene-specific primers for AKT1, mTOR, ERK1/2, JNK1, and GAPDH (housekeeping control, Table 1). Reactions were performed in triplicate in a 10 - 20 μL volume under standard cycling conditions (initial denaturation at 95°C for 2 minutes, followed by 40 cycles of 95°C for 15 seconds and 60°C for 60 seconds). Melt-curve analysis was conducted to confirm single-product amplification. Data were analyzed using the 2^-ΔΔCt^ method (15).

**Table 1.** Primer Sequences Used for Quantitative Real-time Polymerase Chain Reaction

Genes	Forward (5′→3′)	Reverse (5′→3′)
**AKT**	TGGACTACCTGCACTCGGAGAA	GTGCCGCAAAAGGTCTTCATGG
**mTOR **	AGCATCGGATGCTTAGGAGTGG	CAGCCAGTCATCTTTGGAGACC
**ERK1/2**	TGGCAAGCACTACCTGGATCAG	GCAGAGACTGTAGGTAGTTTCGG
**JNK1 **	GACGCCTTATGTAGTGACTCGC	TCCTGGAAAGAGGATTTTGTGGC
**GAPDH **	GTGGTCTCCTCTGACTTCAACA	CTCTTCCTCTTGTGCTCTTGCT

Abbreviations: AKT, protein kinase B; mTOR, mechanistic target of rapamycin; ERK1/2, extracellular signal-regulated kinase 1/2; JNK1, N-terminal kinase.

### 3.8. Enzyme-Linked Immunosorbent Assay for Tumor Necrosis Factor-Alpha and Interleukin-1 Beta

Supernatants were analyzed using ELISA kits for human TNF-α (Elabscience, Cat. No. E-EL-H0109) and IL-1β (Elabscience, Cat. No. E-EL-H0149) according to the manufacturer’s instructions. Absorbance was measured at 450 nm using a microplate reader (BioTek Synergy HTX), and cytokine concentrations were calculated from standard curves and expressed as pg/mL.

### 3.9. Western Blot Analysis

Protein lysates were prepared using RIPA buffer (Thermo Fisher Scientific, Cat. No. 89900) and quantified using the BCA Protein Assay Kit (Thermo Fisher Scientific, Cat. No. 23227). Thirty micrograms (30 μg) of total protein per sample were separated by SDS-PAGE using 10% polyacrylamide gels and transferred to PVDF membranes (Millipore, Cat. No. IPVH00010). Membranes were blocked with 5% non-fat dry milk in TBST and incubated overnight at 4°C with the following primary antibodies: The p-AKT (Ser473, Cell Signaling Technology, Cat. No. 9271), AKT (Cell Signaling Technology, Cat. No. 9272), p-ERK1/2 (Thr202/Tyr204, Cell Signaling Technology, Cat. No. 9101), ERK1/2 (Cell Signaling Technology, Cat. No. 9102), mTOR (Abcam, Cat. No. ab2732), BAX (Abcam, Cat. No. ab32503), BCL-2 (Abcam, Cat. No. ab182858), cleaved caspase-3 (Cell Signaling Technology, Cat. No. 9661), total caspase-3 (Cell Signaling Technology, Cat. No. 9662), and GAPDH (Santa Cruz Biotechnology, Cat. No. sc-47724).

After washing, membranes were incubated with horseradish peroxidase (HRP)-conjugated secondary antibodies (anti-rabbit IgG, Cell Signaling Technology, Cat. No. 7074; anti-mouse IgG, Cell Signaling Technology, Cat. No. 7076) for 1 hour at room temperature. Protein bands were visualized using ECL substrate (Thermo Fisher Scientific, Cat. No. 32106) and imaged with a ChemiDoc XRS+ system (Bio-Rad). Densitometric analysis was performed using ImageJ software (NIH, USA), and band intensities were normalized to GAPDH as the internal control.

### 3.10. Ethical Statement

This study utilized only commercially available human thyroid carcinoma cell lines (TPC-1, ATCC CRL-9520). No human participants or animal models were involved; therefore, institutional ethical approval was not required.

### 3.11. Statistical Analysis

All experiments were performed in triplicate (n = 3). Data are expressed as mean ± SD. Statistical analysis was conducted using one-way analysis of variance (ANOVA) followed by Tukey’s post-hoc test (GraphPad Prism 8). A P-value < 0.05 was considered statistically significant.

## 4. Results

### 4.1. Effects of Bavachinin on Cell Viability and Morphological Changes in TPC-1 Cells

**Figure 1.**
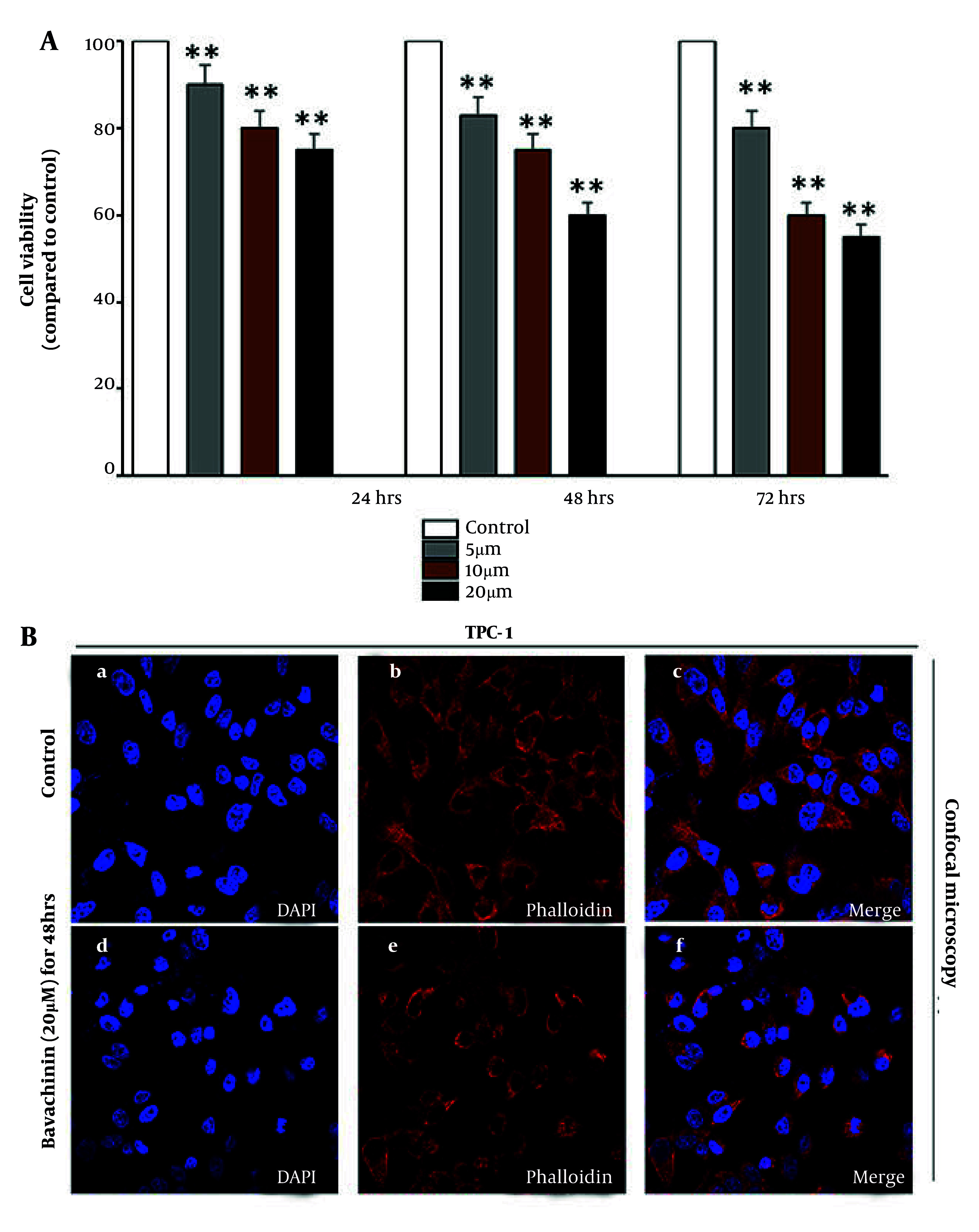
Effects of bavachinin on cell viability and morphological changes in TPC-1 cells. A, TPC-1 cells were treated with 5, 10, and 20 μM bavachinin for 24, 48, and 72 hours, and cell viability was measured by MTT assay. Absorbance was recorded at 570 nm and expressed as percentage of control [mean ± standard deviation (SD), n = 3 independent experiments]. B, TPC-1 cells cultured on coverslips were treated with 20 μM bavachinin for 48 hours. Cells were fixed with 4% paraformaldehyde, permeabilized with 0.1% Triton X-100, and stained with DAPI (blue) and phalloidin-Alexa Fluor 594 (red). Images were acquired using confocal laser scanning microscopy (63× magnification; ** P < 0.01 versus control).

To evaluate the dose- and time-dependent cytotoxic effects of bavachinin on TPC-1 thyroid cancer cells and to examine morphological alterations in cell architecture following treatment, cell viability was assessed by MTT assay at 24, 48, and 72 hours after treatment with 5, 10, and 20 μM bavachinin (Figure 1A). In control (untreated) TPC-1 cells, viability remained at approximately 100% across all time points. Bavachinin treatment led to a significant, dose-dependent reduction in cell viability. At 24 hours, cell viability decreased to approximately 90%, 80%, and 70% for 5, 10, and 20 μM treatments, respectively. This inhibitory effect was more pronounced at 48 and 72 hours, with viability further reduced to 85%, 70%, and 60% at 48 hours and 80%, 60%, and 50% at 72 hours, respectively. These data indicate that bavachinin exerts a time-dependent cytotoxic effect, with higher concentrations yielding more pronounced inhibition of cell viability in TPC-1 cells.

Morphological changes in TPC-1 cells were visualized by confocal microscopy after 48 hours of bavachinin (20 μM) treatment (Figure 1B). DAPI staining (blue) labeled nuclei, while phalloidin (red) labeled the F-actin cytoskeleton. In control cells, nuclei appeared intact and uniformly stained, and the actin cytoskeleton was well-organized, with prominent stress fibers and a spread morphology characteristic of healthy, adherent cells (upper panel). Upon treatment with 20 μM bavachinin, cells displayed marked morphological changes associated with apoptosis. DAPI staining revealed nuclear condensation and fragmentation, while phalloidin-labeled actin filaments exhibited disrupted cytoskeletal architecture, reduced fiber density, and loss of normal cell spreading. Treated cells appeared more rounded and shrunken, indicating cytoplasmic condensation consistent with early apoptotic features (lower panel).

Collectively, these data demonstrate that bavachinin reduces TPC-1 thyroid cancer cell viability in a dose- and time-dependent manner and induces distinct morphological changes consistent with apoptotic cell death. These findings support the hypothesis that bavachinin exerts its anti-cancer effects through induction of apoptosis and provide a foundation for further mechanistic investigations.

### 4.2. Bavachinin Inhibits Migration and Invasion in TPC-1 Thyroid Cancer Cells

**Figure 2.**
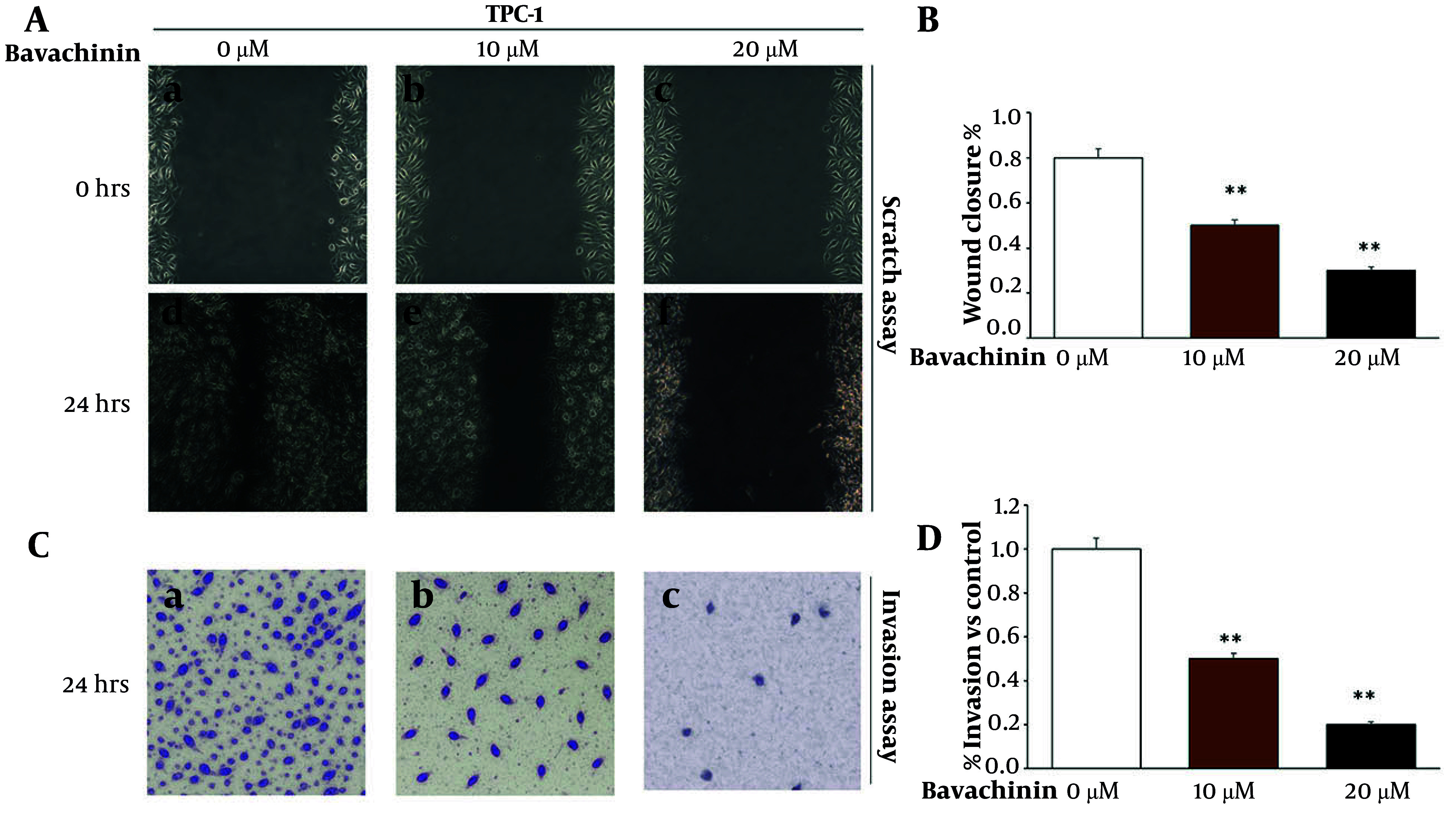
Bavachinin inhibits migration and invasion in TPC-1 cells. A, wound healing assay images were captured at 0 and 24 hours post-scratch in control and bavachinin-treated (10 and 20 μM) TPC-1 cells. B, quantification of percentage wound closure at 24 hours using ImageJ software [mean ± standard deviation (SD), n = 3 independent experiments]. C, transwell invasion assay was conducted for 24 hours with Matrigel-coated inserts. Invaded cells were fixed with methanol and stained with crystal violet. D, quantification of percentage invasion normalized to control (mean ± SD, n = 3 independent experiments; ** P < 0.01 versus control).

The effect of bavachinin on the migratory and invasive capabilities of TPC-1 cells was evaluated using wound healing and Transwell invasion assays. As shown in Figure 2A, control cells exhibited significant wound closure after 24 hours, consistent with their inherent migratory capacity. In contrast, bavachinin treatment resulted in a clear dose-dependent inhibition of migration, with 10 μM and 20 μM treatments markedly reducing wound area closure compared to control. Quantitative analysis of these images (Figure 2B) demonstrated that control cells achieved approximately 95% wound closure, whereas 10 μM bavachinin treatment reduced this to 55% and 20 μM bavachinin further reduced migration to 23%.

To further assess the effect of bavachinin on cell invasiveness, a Transwell invasion assay was performed at 24 hours (Figure 2C). In the control group, a high number of TPC-1 cells successfully invaded through the Matrigel-coated membrane, while treatment with 10 μM and 20 μM bavachinin markedly decreased the number of invaded cells in a dose-dependent manner. Quantitative analysis (Figure 2D) confirmed this trend, with invasion rates decreasing from 100% in control to 55% and 20% in the 10 μM and 20 μM treated groups, respectively.

These findings indicate that bavachinin significantly inhibits both migration and invasion in TPC-1 thyroid cancer cells, suggesting that its anti-cancer effects extend beyond cytotoxicity to include disruption of key mechanisms underlying tumor metastasis.

### 4.3. Bavachinin Suppresses Phosphoinositide-3-kinase/AKT/Mechanistic Target of Rapamycin and Mitogen-Activated Protein Kinase/Extracellular Signal-Regulated Kinase Signaling in TPC-1 Cells

**Figure 3.**
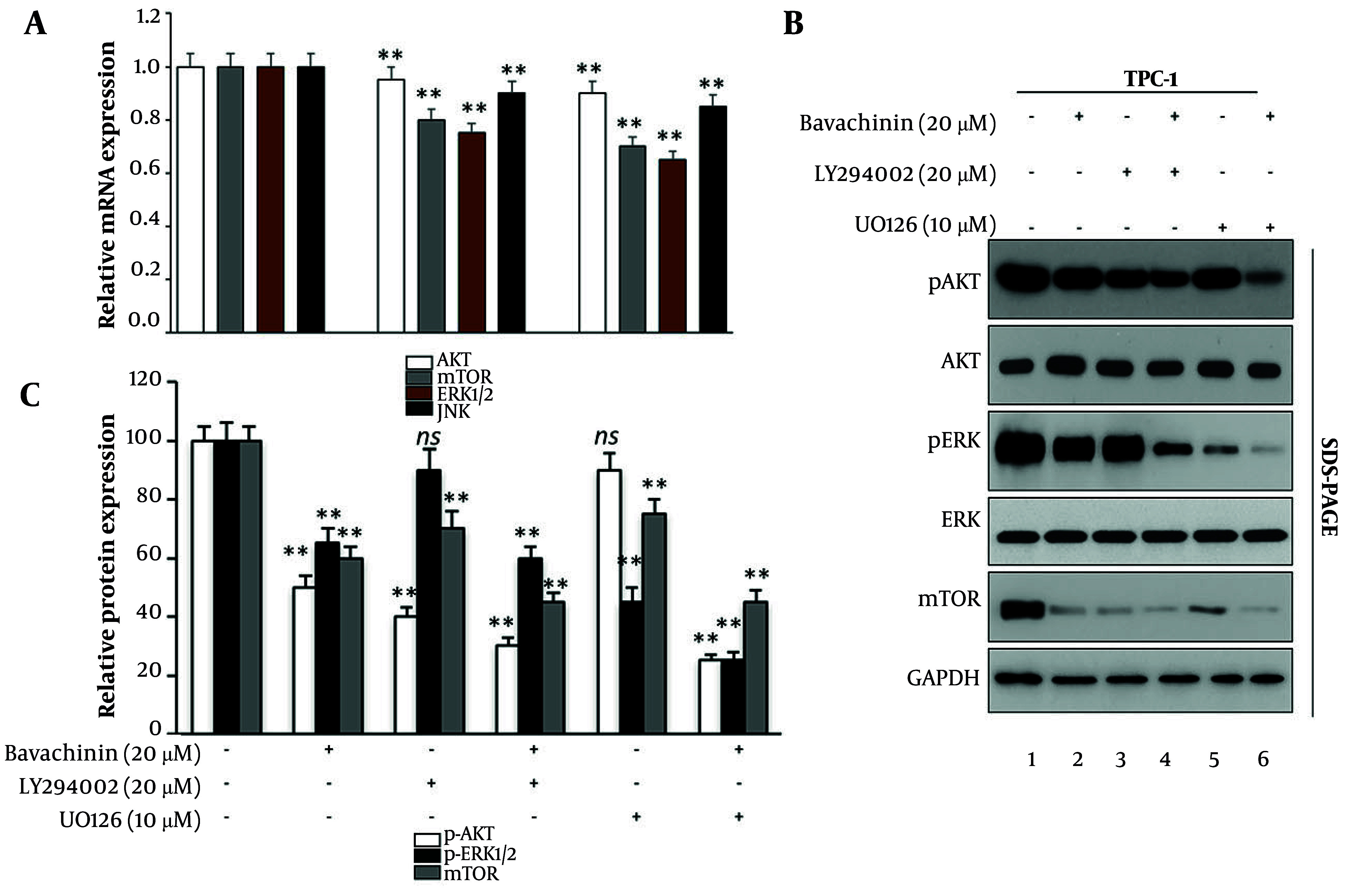
Bavachinin suppresses phosphoinositide-3-kinase (PI3K)/AKT/mechanistic target of rapamycin (mTOR) and mitogen-activated protein kinase (MAPK)/extracellular signal-regulated kinase (ERK) signaling in TPC-1 cells. A, quantitative real-time polymerase chain reaction (qRT-PCR) analysis of AKT, mTOR, ERK1/2, and N-terminal kinase (JNK) mRNA expression in control and bavachinin-treated (10 and 20 μM) TPC-1 cells, normalized to GAPDH [mean ± standard deviation (SD), n = 3 independent experiments]. B, representative Western blot analysis of p-AKT, total AKT, p-ERK1/2, total ERK1/2, mTOR, and GAPDH across six treatment groups: Control, bavachinin (20 μM), LY294002, LY294002 + bavachinin, U0126, and U0126 + bavachinin. Protein bands were detected by enhanced chemiluminescence. C, densitometric quantification of Western blots showing relative expression of p-AKT/AKT, p-ERK/ERK, and mTOR, expressed as a percentage of control (mean ± SD, n = 3 independent experiments; ** P < 0.01 versus control).

To elucidate the molecular mechanisms underlying bavachinin’s effects, we analyzed the PI3K/AKT/mTOR and MAPK/ERK pathways, both central to thyroid cancer progression. As shown in Figure 3A, qRT-PCR revealed that treatment with bavachinin (10 and 20 μM) significantly reduced mRNA expression of AKT, mTOR, ERK1/2, and JNK, indicating transcriptional downregulation of key oncogenic drivers.

Western blot analysis across six treatment groups — control, bavachinin (20 μM), LY294002 (PI3K inhibitor), LY294002 + bavachinin, U0126 (MAPK inhibitor), and U0126 + bavachinin — further validated these effects (Figure 3B). Bavachinin alone suppressed p-AKT to approximately 50% and p-ERK1/2 to approximately 65% of control without altering total protein levels. Notably, combining bavachinin with LY294002 enhanced p-AKT suppression to approximately 30% of control, while bavachinin with U0126 reduced p-ERK1/2 to approximately 25% of control. Downstream, mTOR protein was also reduced to approximately 60% by bavachinin alone and approximately 45% in the bavachinin + U0126 group. Figure 3C provides densitometric analysis.

These results demonstrate that bavachinin acts as a dual-arm inhibitor of PI3K/AKT/mTOR and MAPK/ERK signaling in thyroid carcinoma cells. Furthermore, its combination with pathway-specific inhibitors produces additive suppression, underscoring bavachinin’s unique ability to simultaneously target both signaling axes. This dual inhibition distinguishes bavachinin’s activity in thyroid carcinoma from its previously reported effects in other cancer models.

### 4.4. Bavachinin Reduces Pro-Inflammatory Cytokine Secretion in TPC-1 Cells

**Figure 4.**
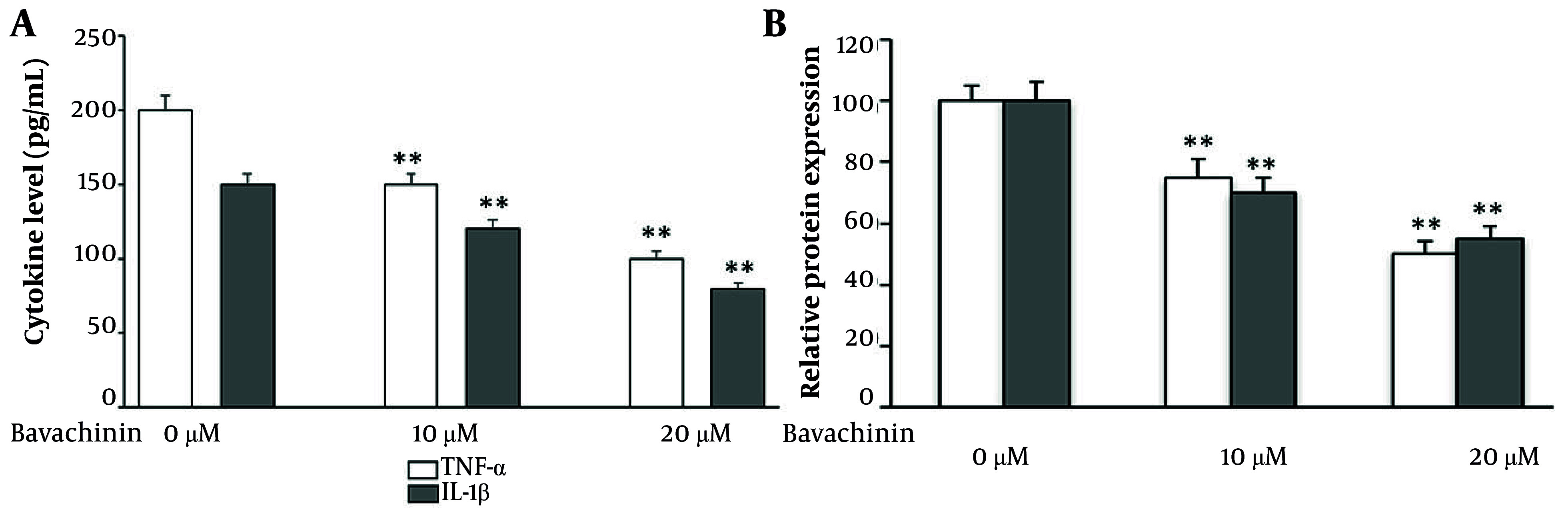
Bavachinin attenuates pro-inflammatory cytokines in TPC-1 cells. A, concentrations of tumor necrosis factor-alpha (TNF-α) and interleukin-1 beta (IL-1β) in culture supernatants of TPC-1 cells treated with vehicle (control) or bavachinin (10 and 20 μM) for 24 hours, measured by enzyme-linked immunosorbent assay [ELISA, mean ± standard deviation (SD), n = 3 independent experiments\. B, cytokine levels expressed as a percentage of control, summarizing dose-dependent suppression of TNF-α and IL-1β after bavachinin treatment (mean ± SD, n = 3; ** P < 0.01 versus control).

To determine whether bavachinin modulates the inflammatory microenvironment, we quantified secreted cytokines in TPC-1 culture supernatants following 24-hour treatment. As shown in Figure 4A, bavachinin induced a clear, dose-dependent reduction in both TNF-α and IL-1β. TNF-α secretion declined from approximately 200 pg/mL in control cells to approximately 150 pg/mL at 10 μM and approximately 100 pg/mL at 20 μM, while IL-1β levels decreased from approximately 150 pg/mL in control to approximately 105 pg/mL at 10 μM and approximately 80 pg/mL at 20 μM. To summarize these changes, cytokine concentrations were expressed as a percentage of control (Figure 4B). At 20 μM bavachinin, TNF-α and IL-1β secretion were suppressed to approximately 50% and approximately 55% of baseline, respectively.

These findings confirm that bavachinin not only inhibits oncogenic signaling pathways (Figure 3) but also attenuates pro-inflammatory cytokines, highlighting a dual mechanism of action targeting both cancer cell survival pathways and the tumor-promoting inflammatory milieu.

### 4.5. Bavachinin Induces Apoptosis in TPC-1 Cells

**Figure 5.**
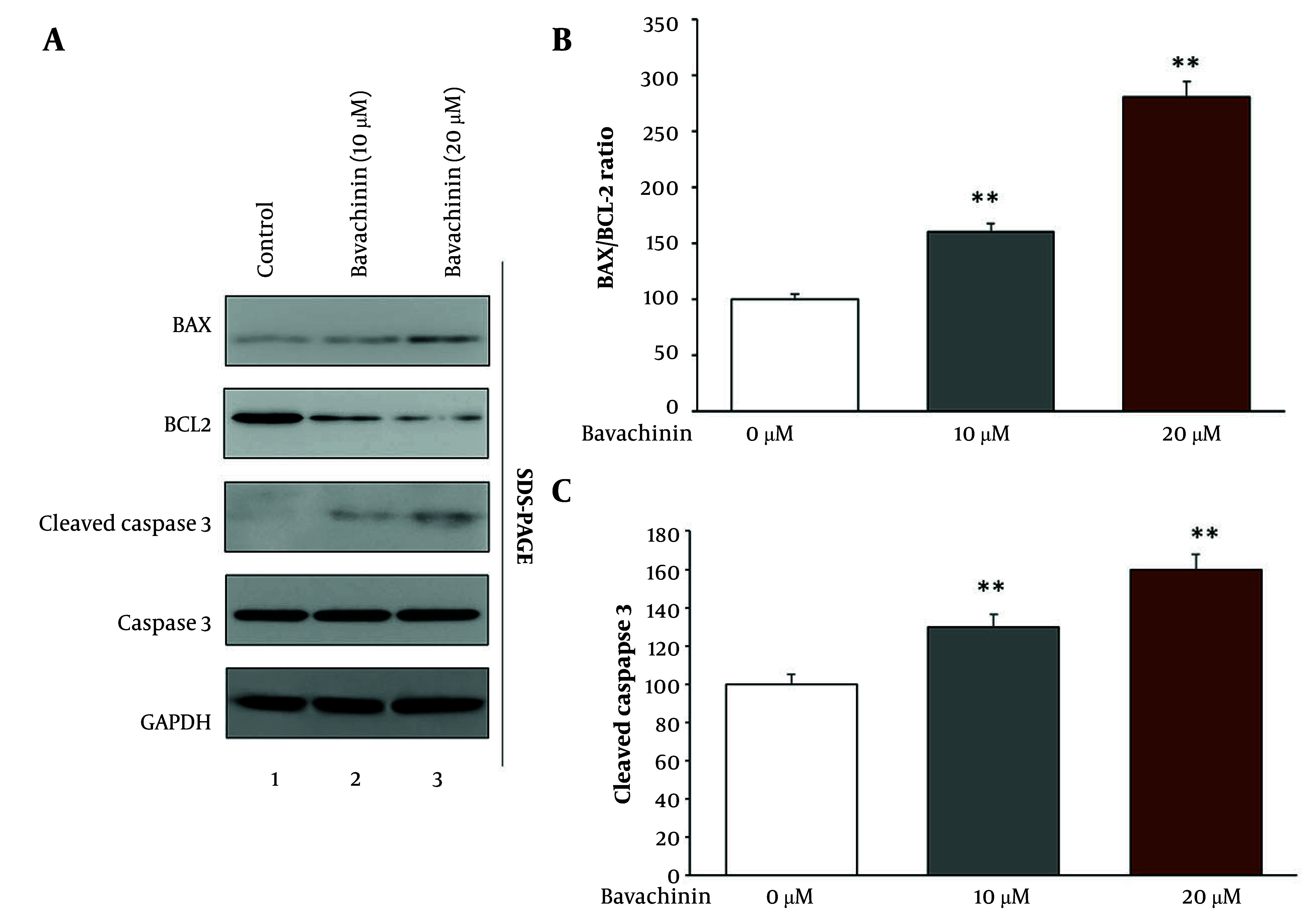
Bavachinin induces apoptosis markers in TPC-1 cells. A, Western blot analysis of B-cell lymphoma-2-associated X protein (BAX), B-cell lymphoma-2 (BCL-2), cleaved caspase-3, total caspase-3, and GAPDH in control and bavachinin-treated (10 and 20 μM) TPC-1 cells. B, densitometric analysis of BAX/BCL-2 protein ratio expressed as percentage of control [mean ± standard deviation (SD), n = 3 independent experiments]. C, densitometric analysis of cleaved caspase-3 protein levels using ImageJ, normalized to total caspase-3 (mean ± SD, n = 3 independent experiments; ** P < 0.01 versus control).

To investigate the pro-apoptotic effects of bavachinin in TPC-1 thyroid cancer cells, we evaluated key markers of apoptosis. Figure 5A shows Western blot analysis of BAX, BCL-2, and cleaved caspase-3 proteins after 48 hours of treatment with control, 10 μM, and 20 μM bavachinin. Bavachinin treatment led to a dose-dependent increase in pro-apoptotic BAX protein levels (130% and 170% of control for 10 and 20 μM, respectively) and a decrease in anti-apoptotic BCL-2 levels (80% and 60% of control, respectively). Similarly, cleaved caspase-3 protein expression increased significantly in bavachinin-treated cells, reaching 130% and 160% of control for 10 and 20 μM doses, respectively. Total caspase-3 and GAPDH levels remained unchanged, serving as loading controls.

Figure 5B quantifies the BAX/BCL-2 protein ratio, revealing a significant, dose-dependent increase from 100% in control to 160% and 280% in the 10 and 20 μM bavachinin groups, respectively. This ratio is a well-established marker of apoptosis induction. Consistently, the caspase-3 activity assay (Figure 5C) showed a robust increase in caspase-3 enzymatic activity, with activity levels reaching 130% and 160% of control in cells treated with 10 and 20 μM bavachinin, respectively. These data collectively indicate that bavachinin activates the intrinsic mitochondrial apoptotic pathway, promoting caspase-mediated cell death in TPC-1 thyroid cancer cells.

### 4.6. Bavachinin Exerts Multi-Targeted Effects Leading to Apoptosis in TPC-1 Cells

**Figure 6.**
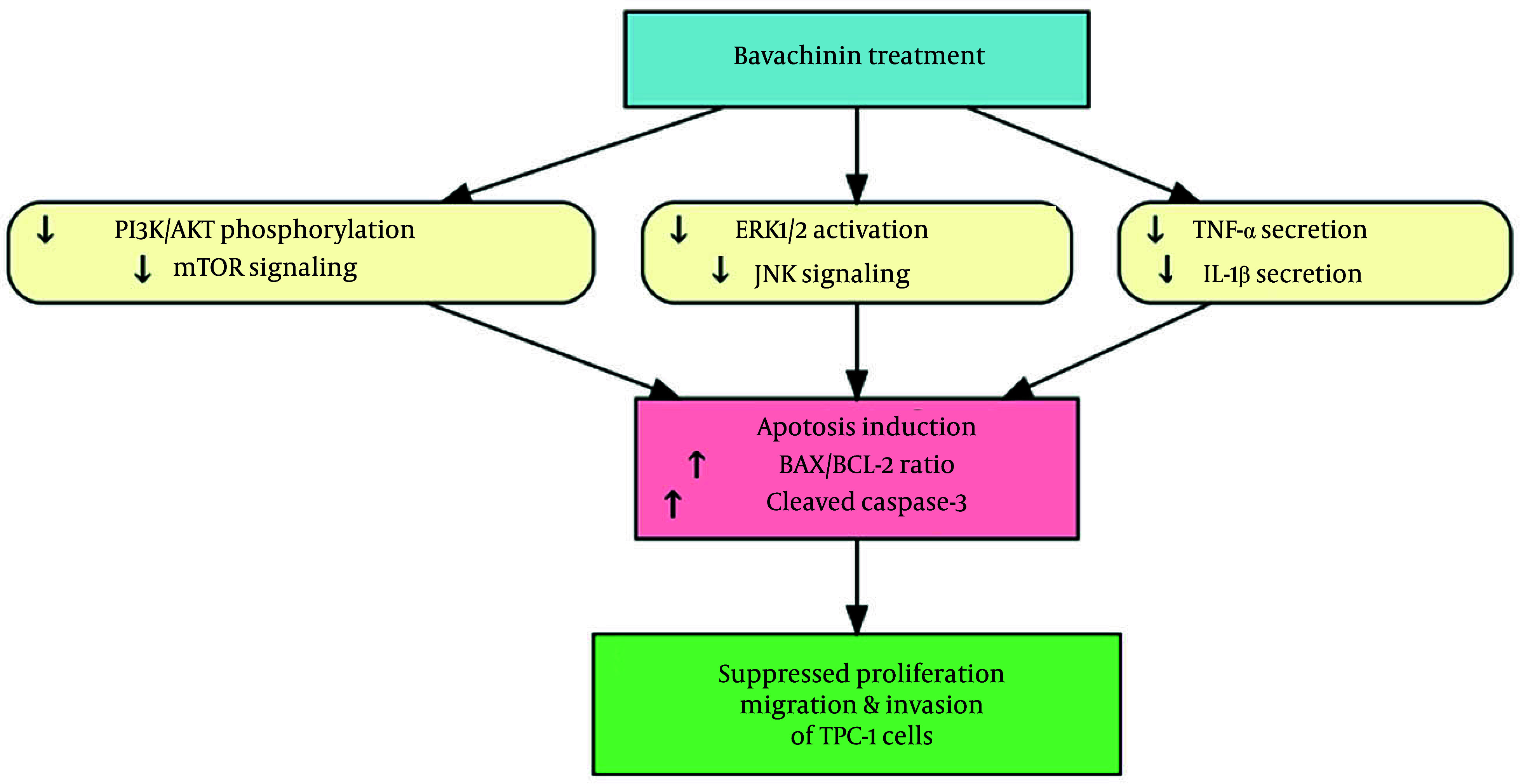
Mechanistic summary of bavachinin’s anti-cancer effects in TPC-1 cells; schematic illustration depicting the multi-targeted effects of bavachinin. Treatment with bavachinin suppresses the phosphoinositide-3-kinase (PI3K)/AKT/mechanistic target of rapamycin (mTOR) and mitogen-activated protein kinase (MAPK)/extracellular signal-regulated kinase (ERK) signaling pathways while concurrently reducing secretion of the pro-inflammatory cytokines tumor necrosis factor-alpha (TNF-α) and interleukin-1 beta (IL-1β). These combined effects converge to promote apoptosis, characterized by an increased B-cell lymphoma-2-associated X protein (BAX)/B-cell lymphoma-2 (BCL-2) ratio and enhanced caspase-3 cleavage, ultimately leading to inhibition of proliferation, migration, and survival of papillary thyroid carcinoma cells.

To integrate the observed molecular and functional effects of bavachinin, a schematic model was constructed (Figure 6). Bavachinin treatment simultaneously inhibited two major oncogenic pathways, PI3K/AKT/mTOR and MAPK/ERK, as demonstrated by reduced phosphorylation of AKT and ERK1/2, along with downregulation of mTOR and JNK. In parallel, bavachinin significantly decreased secretion of the pro-inflammatory cytokines TNF-α and IL-1β, as shown in Figure 4. The convergence of these signaling and cytokine-suppressive effects promoted apoptosis, evidenced by an increased BAX/BCL-2 ratio and enhanced cleavage of caspase-3. Collectively, these findings suggest that bavachinin exerts multi-targeted anti-cancer activity in papillary thyroid carcinoma cells by coordinately suppressing proliferative signaling and inflammatory cytokines, ultimately leading to apoptosis and reduced tumor-promoting potential.

## 5. Discussion

In this study, we demonstrated that bavachinin, a natural flavonoid derived from *Psoralea corylifolia*, exerts potent anti-thyroid cancer effects in TPC‑1 cells by targeting multiple oncogenic pathways, inhibiting migration and invasion, reducing pro-inflammatory cytokine production, and promoting apoptosis via intrinsic mechanisms.

Bavachinin has previously been shown to modulate diverse signaling pathways in other cancer models, including p53/BCL-2/BAX signaling in DMH-induced colon cancer (16), inhibition of the cholesterol-synthesis enzyme FDFT1 via the AKT/mTOR/SREBP-2 pathway (17), and G2/M cell-cycle arrest through p38/p21^Waf1^/Cip1 activation in non-small-cell lung cancer (SCLC) (13). A comprehensive review of bavachinin and its derivatives as multi-therapeutic agents is also available (18). However, none of these studies examined bavachinin in thyroid carcinoma or demonstrated its dual inhibition of the PI3K/AKT/mTOR and MAPK/ERK pathways, highlighting the novelty of our findings.

We found that bavachinin significantly reduced p-AKT and p-ERK1/2 protein levels and simultaneously downregulated mRNA and protein expression of AKT, mTOR, ERK1/2, and JNK. This dual suppression aligns with the therapeutic strategy of targeting both PI3K/AKT/mTOR and MAPK/ERK pathways, which are often co-activated in thyroid cancers — especially in aggressive subtypes — and contribute to tumor survival, proliferation, and therapeutic resistance (19-21). Studies in anaplastic thyroid carcinoma have shown similar benefits in inhibiting both pathways, reinforcing our findings (22).

Moreover, previous research in other cancer models, such as vitamin C treatment in thyroid cancer cells, also supports simultaneous downregulation of these pathways leading to cytotoxicity (23-25). However, our study is the first to report this dual-targeting mechanism specifically mediated by bavachinin in papillary thyroid carcinoma cells.

The ELISA results demonstrated dose-dependent decreases in TNF‑α and IL‑1β secretion. Chronic inflammation enhances thyroid cancer progression, with TNF‑α and IL‑1β known to promote tumor invasiveness and survival (26). By attenuating these cytokines, bavachinin may disrupt this supportive microenvironment. Few botanical compounds targeting these cytokines in thyroid cancer have been reported, making our findings novel and significant.

We observed substantial reductions in cell migration and invasion in wound healing and Transwell assays, echoing previous findings in anaplastic carcinoma cell lines where dual inhibition of PI3K/AKT and MAPK was necessary to impair invasiveness (27). Our results further extend these observations to the suppression of functional cancer traits in papillary carcinoma, reinforcing bavachinin’s therapeutic potential.

Bavachinin induced a robust pro-apoptotic response, characterized by an increased BAX/BCL‑2 ratio, enhanced cleaved caspase‑3 activity, and morphological features of apoptosis, such as chromatin condensation. These molecular signatures reflect intrinsic apoptosis activation. Similar effects of bavachinin in SCLC, where increased BAX and caspase‑3 levels were reported, support the conserved pro-apoptotic action of this compound (8, 9, 19). Notably, while bavachinin’s effects on cell cycle arrest via ATM/ATR pathways were reported in SCLC (19), our data reveal a thyroid-specific apoptotic mechanism mediated via signaling inhibition and caspase activation.

Recent studies further support the therapeutic relevance of apoptosis-inducing natural compounds in cancer therapy. For instance, steviol glycoside has been shown to trigger apoptosis in MCF-7 and A2780 cell lines, demonstrating mechanistic parallels with bavachinin (28). Additionally, a case report describing lenvatinib-induced posterior reversible encephalopathy syndrome in a papillary thyroid carcinoma patient highlights the toxicity challenges of existing kinase inhibitors, underscoring the need for natural agents such as bavachinin with potentially safer pharmacological profiles (29).

The present schematic (Figure 6) encapsulates these multi-level effects, highlighting a master regulatory model in which bavachinin suppresses survival pathways, diminishes inflammatory signals, and triggers apoptosis, leading to reduced cell viability and invasiveness. With mechanistic parallels to vitamin C’s dual pathway inhibition in thyroid cancer, yet distinct mediating agents, bavachinin emerges as a distinct and targeted anti-neoplastic compound.

A limitation of this study is the absence of experiments using normal human thyroid epithelial cells as a control group, which would help confirm tumor selectivity. Future work will include such comparisons to strengthen translational relevance and evaluate bavachinin’s selectivity toward malignant versus non-malignant thyroid cells.

While our in vitro data strongly support bavachinin’s efficacy, in vivo validation remains a crucial next step, given its positive outcomes in other models such as SCLC xenografts. Future studies should also evaluate bavachinin’s effects in other thyroid cancer subtypes and investigate potential synergistic effects with existing kinase inhibitors or chemotherapy, especially in aggressive tumors harboring dual pathway activations (e.g., BRAF/PTC or RAS mutations).

### 5.1. Conclusions

In conclusion, bavachinin exerts multi-targeted anti-cancer effects in papillary thyroid carcinoma cells by modulating PI3K/AKT/mTOR and MAPK/ERK pathways, suppressing pro-inflammatory cytokines, and inducing apoptosis. These findings identify bavachinin as a potential candidate, pending in vivo validation, for further exploration in thyroid cancer therapy.

## Data Availability

The data presented in this study are uploaded during submission as a supplementary file and are openly available for readers upon request.
